# Taxonomic studies on genus *Tetramorium* Mayr (Hymenoptera, Formicidae) with report of two new species and three new records including a tramp species from India with a revised key

**DOI:** 10.3897/zookeys.207.3040

**Published:** 2012-07-11

**Authors:** Himender Bharti, Rakesh Kumar

**Affiliations:** 1 Department of Zoology & Environmental Sciences, Punjabi University, Patiala, Punjab- 147002, India

**Keywords:** *Tetramorium*, Myrmicinae, new species, tramp species, India, key

## Abstract

Two new species of *Tetramorium* Mayr, namely *Tetramorium shivalikense* **sp. n.** and *Tetramorium triangulatum* **sp. n.** are described.* Tetramorium triangulatum* **sp. n.** belongs to the *inglebyi*-species group and is described based on worker, queen and male caste, while *Tetramorium shivalikense* **sp. n.** belongs to the *ciliatum*-species group and is described based on worker caste only. Three species viz., *Tetramorium caldarium* (Roger), *Tetramorium tonganum* Mayr and *Tetramorium urbanii* Bolton represent first records from India. The male caste is described for the first time in the case of *Tetramorium tonganum*. Among these, *Tetramorium caldarium* is a tramp species which extends its limit to India as well. A revised key to the Indian ants of this genus is also provided herewith.

## Introduction

*Tetramorium* Mayr, 1855 is one of the largest genera within the tribe Tetramoriini and is one of the most species rich genera with 477 species (Bolton 2012). The genus has a worldwide distribution with varying species richness among different zoogeographical regions. The greatest number of species (230) has been reported from the Afrotropical region, whereas there are only very few (13) known from the New World. The genus has a good representation in the Palaearctic, Oriental, Malagasy and Indo-Australian regions, although much less compared to the Afrotropical region (Hita Garcia and Fisher 2011). Bolton (1976, 1977, 1979, 1980 and 1985) revised this genus for most of the above stated regions except the Palaearctic, and noteworthy contributions for the Palaearctic include Steiner et al. (2005), Schlick-Steiner et al. (2006), Csösz et al. (2007), Csösz and Schulz (2010), Steiner et al. (2010). Other significant contributions to this genus dealing with revision of species groups and complexes from the Palaearctic, Afrotropical and Malagasy regions are Csösz et al. (2007), Csösz and Schulz (2010), Hita Garcia et al. (2010), Hita Garcia and Fisher (2011).

In India, the genus *Tetramorium* is currently represented by 30 species (Bharti 2011). Most of the Indian species were treated by Bolton (1976, 1977) in his revisionary work dealing with the Oriental and Indo-Australian regions. Later, the contributions which reported new species included Mathew (1981), Sheela and Narendran (1998), Mathew and Tiwari (2000), and Bharti (2011) who provided a replacement name for *Tetramorium browni* Tiwari. Fourteen species out of 30 described hitherto have been reported from Southern India, 3 from North-eastern India, 2 from North India, 1 from Central India. Five Indian species *Tetramorium indicum* Forel, *Tetramorium obesum* Andre, *Tetramorium smithi* Mayr, *Tetramorium tortuosum* Roger and *Tetramorium walshi* (Forel) have wide distribution ranges in South-East Asia. Five tramp species namely *Tetramorium bicarinatum* (Nylander), *Tetramorium caespitum* (Linnaeus), *Tetramorium lanuginosum* Mayr, *Tetramorium pacificum* Mayr, *Tetramorium simillimum* (Smith) are widely distributed in India as well. Thus, the genus is mainly known from the southern region of the country. During the present study two new species *Tetramorium triangulatum* sp. n. and *Tetramorium shivalikense* sp. n. are described from the northern part of the country. The three species *Tetramorium caldarium* (Roger), *Tetramorium tonganum* Mayr and *Tetramorium urbanii* Bolton represent first records from India. Moreover, *Tetramorium caldarium* is a well established tramp species and *Tetramorium tonganum* a probable tramp species. Male caste of *Tetramorium tonganum* is described for the first time.

With the addition of 5 species, the genus *Tetramorium* is now represented by 35 species from India. These species are placed in the following 12 species groups except *Tetramorium beesoni* (Mukerjee) for which the group is unknown: *angulinode*-group: *Tetramorium smithi* Mayr; *bicarinatum*-group: *Tetramorium bicarinatum* (Nylander), *Tetramorium indicum* Forel, *Tetramorium pacificum* Mayr, *Tetramorium meghalayense* Bharti, *Tetramorium petiolatum* Sheela and Narendran; *caespitum*-group: *Tetramorium caespitum* (Linnaeus); *ciliatum*-group: *Tetramorium shivalikense* sp. n.; *fergusoni*-group: *Tetramorium fergusoni* Forel; *inglebyi*-group: *Tetramorium elisabethae* Forel, *Tetramorium inglebyi* Forel, *Tetramorium myops* Bolton, *Tetramorium triangulatum* sp. n.; *mixtum*-group: *Tetramorium mixtum* Forel, *Tetramorium rugigaster* Bolton, *Tetramorium malabarense* Sheela and Narendran, *Tetramorium sentosum* Sheela and Narendran; *obesum*-group: *Tetramorium coonoorense* Forel, *Tetramorium decamerum* (Forel), *Tetramorium lanuginosum* Mayr, *Tetramorium obesum* Andre, *Tetramorium rossi* (Bolton); *simillimum*-group: *Tetramorium simillimum* (Smith), *Tetramorium caldarium* (Roger); *tonganum*-group: *Tetramorium christiei* Forel, *Tetramorium salvatum* Forel, *Tetramorium tonganum* Mayr, *Tetramorium barryi* Mathew; *tortuosum*-group: *Tetramorium belgaense* Forel, *Tetramorium tortuosum* Roger, *Tetramorium urbanii* Bolton, *Tetramorium keralense* Sheela and Narendran; *walshi*-group: *Tetramorium cordatum* Sheela and Narendran, *Tetramorium walshi* (Forel). A revised key to the Indian ants of this genus is also provided herewith.

## Materials and methods

The ants were collected by pitfall traps, hand picking, soil core sampling, beating vegetation, and from the leaf litter with Winkler’s extractor. The digital images of these specimens were prepared on a Nikon SMZ-1500 stereo zoom microscope using Auto-Montage software. Later, images were cleaned with Adobe Photoshop CS5.

Abbreviations of the type depositories are as follows: BMNH, The Natural History Museum, London, U.K.; PUPAC, Punjabi University Patiala Ant Collection, Patiala, India. Two paratypes of each, *Tetramorium shivalikense* sp. n. and *Tetramorium triangulatum* sp. n.,will be deposited in BMNH.

Measurements and indices follow Hita Garcia and Fisher (2011): head Length (HL), head Width (HW), scape Length (SL), eye length (EL), pronotal width (PW), weber’s length (WL), propodeal spine Length (PSL), petiolar node height (PTH), petiolar node length (PTL), petiolar node width (PTW), postpetiole height (PPH), postpetiole length (PPL), postpetiole width (PPW), ocular index (OI), cephalic index (CI), scape index (SI), propodeal spine index (PSLI), petiolar node index (PeNI), lateral petiole index (LPeI), dorsal petiole index (DPeI), postpetiolar node index (PpNI) = PPW/PW*100, lateral postpetiole index (LPpI), dorsal postpetiole index (DPpI), postpetiole index (PPI).

## Results

### Tetramorium shivalikense

sp. n.

urn:lsid:zoobank.org:act:C846D381-9691-4465-8098-FF44357F415C

http://species-id.net/wiki/Tetramorium_shivalikense

Figs 1–3

#### Holotype.

Worker, India, Himachal Pradesh, Terrace, 31.928591°N, 75.931342°E, 420m alt., winkler, 11 October 2008, coll. R. Kumar, PUPAC.

#### Paratypes.

11(w), India, Himachal Pradesh, Terrace, 420m alt., winkler, 11 October 2008; 1(w), India, Punjab, Dharampur, 450m alt., beating, 14 October 2008; 18(w), India, Himachal Pradesh, Siholi, 550m alt., winkler, 19 October 2008; 1(w), India, Himachal Pradesh, Terrace, 420m alt., winkler, 25 May 2009; 1(w), India, Uttarakhand, Dehradun, Selaqui, 650m alt., 9 August 2009, winkler; 50(w), India, Uttarakhand, Dehradun, Forest Research Institute, 640m alt., hand picking, 14 August 2009; 1(w), India, Uttarakhand, Dehradun, Forest Research Institute, 640m alt., winkler, 17 August 2009; 1(w), India, Himachal Pradesh, Terrace, 420m alt., pitfall trap, 24 September 2009; 4(w), India, Himachal Pradesh, Ghati, 450m alt., winkler, 27 September 2009; 2(w), India, Himachal Pradesh, Chanaur, 600m alt., winkler, 3 October 2009; 2(w), India, Himachal Pradesh, Andretta, 940m alt., winkler, 11June 2010; 8(w), India, Himachal Pradesh, Palampur, 1140m alt., winkler, 14 June 2010; 1(w), India, Himachal Pradesh, Dattal, 940m alt., winkler, 16 June 2010; coll. R. Kumar; PUPAC and two paratypes will be deposited in BMNH.

#### Worker description.

Measurements: Holotype worker. HL 0.56, HW 0.52, SL 0.34, EL 0.13, WL 0.60, PW 0.38, PSL 0.13, PTL 0.14, PPL 0.19, PTW 0.20, PPW 0.23, PTH 0.20, PPH 0.20, CI 92.86, OI 25.00, SI 65.38, PSLI 23.21, PeNI 52.63, LPeI 70.00, DPeI 142.86, PpNI 60.53, LPpI 95.00, DPpI 121.05, PPI 115.00.

Paratype workers. HL 0.56-0.62, HW 0.52-0.56, SL 0.34-0.35, EL 0.13-0.14, WL 0.60-0.67, PW 0.38-0.42, PSL 0.13-0.14, PTL 0.13-0.14, PPL 0.19-0.23, PTW 0.20-0.23, PPW 0.23-0.25, PTH 0.20-0.21, PPH 0.20-0.21, CI 89.83-92.86, OI 24.53-25.00, SI 62.50-66.04, PSLI 22.03-23.21, PeNI 51.22-54.76, LPeI 61.90-70.00, DPeI 142.86-164.29, PpNI 59.52-60.98, LPpI 95.00-109.52, DPpI 108.70-125.00, PPI 108.70-119.05 (10 measured).

Head slightly longer than broad, sides almost straight with rounded posterolateral corners, slightly broader posteriorly than anteriorly; posterior head margin straight with shallow median notch; clypeus convex with steep apical half; anterior margin of clypeus entire without median notch; anterior margin of clypeus with a narrow transverse plate-like fringe and having convex anterior margin; mandibles triangular, masticatory margin of mandibles with large apical and preapical tooth; third tooth slightly smaller than the preapical tooth followed by 3–4 denticles; frontal lobes weakly developed and elevated laterally, frontal area indistinct; antennal scrobes shallow and broad; eye moderate in size, located laterally and at mid-length of head, composed of ca. 35–36 ommatidia; antennae slender, 12-segmented; scape short from posterior head margin by one fourth of its length; mesosoma slightly longer than head, broader anteriorly than posteriorly, dorsum convex; pro-mesonotal suture and metanotal groove indistinct; propodeal spine longer (PSL 0.13–0.14mm), acute, divergent, with tips upcurved; propodeal lobes roughly broadly triangular and acute; posterior declivity of propodeum short, concave; petiole with a short peduncle, node slightly broader than long in dorsal view; base of node longer than dorsal face in lateral view, weakly convex dorsum in lateral view; ventrally petiole downcurved along its length; postpetiole broader than long; base of first gastral tergite weakly concave behind the postpetiole, anterolateral corners rounded and not projecting forward as a pair of blunt teeth or horns which go round the sides of the posterior portion of the postpetiole, gaster oval.

Head longitudinally rugose with few cross-meshes up to vertex, posteriorly reticulate-rugose, interrugal space punctured and shiny; frontal carinae strongly developed and somewhat short to reach posterior head corners; mandibles longitudinally rugulose and interrugal space smooth and shiny; clypeus with a strong median carina continued to vertex and two weak lateral carinae; dorsum of mesosoma reticulate-rugose; sides of mesosoma rugo-reticulate but weaker sculptured than dorsum; dorsum of node with an unsculptured median longitudinal strip and sides with weak rugosity; dorsum of postpetiole smooth and shiny, sides with weak rugosity; propodeal declivity, gaster and legs smooth and shiny.

Body darker brown in most specimens and few specimens yellowish brown; Whole body coveredwith abundant, long, erect and short subdecumbent pilosity; antennal scapes and hind tibiae with short suberect hairs.

#### Etymology.

The specific epithet refers to the collection area.

#### Ecology. 

This new species is widespread in the Shivalik range of the north-western Himalaya and was collected from soil and leaf litter.

#### Remarks.

This new species belongs to the *ciliatum*- species group (Bolton 1977) which is distributed in the Oriental and Indo-Australian regions. The characteristics of this group are: antennae 12-segmented, sting appendage triangular or dentiform, anterior clypeal margin entire and not notched or indented medially, frontal carinae extending well beyond the level of the posterior margins of the eyes, propodeal spines long and usually strongly developed, never downcurved along their length, anterolateral gastral corners not projecting forward as a pair of blunt teeth or horns which go round the sides of the posterior portion of the postpetiole.

*Tetramorium shivalikense* sp. n. is somewhat allied to *Tetramorium zypidum* Bolton. However, it can be easily distinguished from *Tetramorium zypidum* by the following combination of characters: anterior margin of clypeus is entire and convex, petiolar node slightly broader than long in dorsal view while in *Tetramorium zypidum* theanterior clypeal margin is shallowly impressed medially and the petiolar node significantly longer than broad. Other significant characters of *Tetramorium shivalikense* sp. n. which differentiate it from *Tetramorium zypidum* include eyes located laterally at mid-length of the head, propodeal lobes broadly triangular and acute, SI 62.50-66.04, sides of postpetiole with weak rugosity, frontal carinae strongly developed and somewhat short to reach posterior head corners. In *Tetramorium zypidum* the eyes are situated in front of the middle of the sides of the head, the propodeal lobes narrowly triangular and acute, SI 69.00–75.00, sides of postpetiole smooth, and the frontal carinae extend to the posterior head corners and are weak behind the level of the eyes.

**Figures 1–3.**
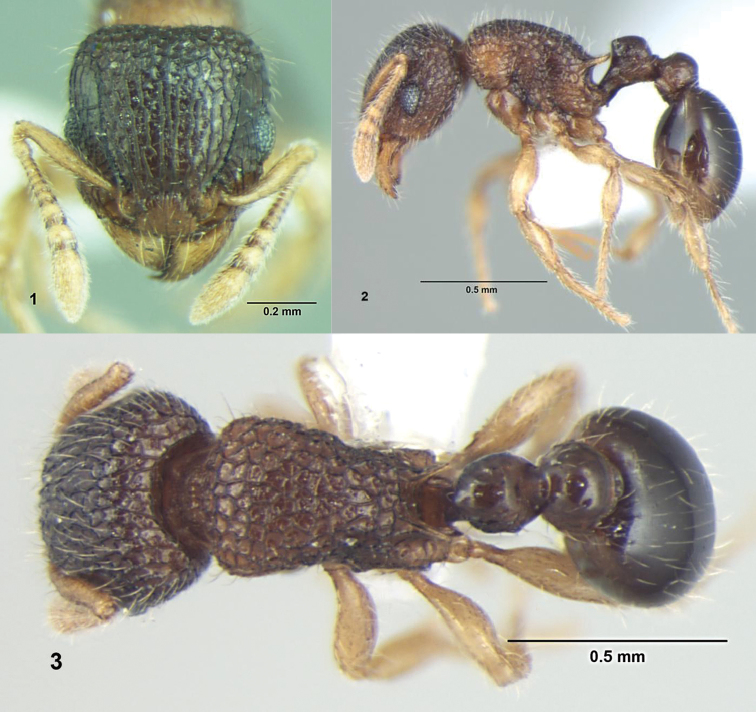
*Tetramorium shivalikense* sp. n., Worker: **1** Head, full-face view **2** Body, lateral view **3** Body, dorsal view.

### Tetramorium triangulatum

sp. n.

urn:lsid:zoobank.org:act:8552E147-02EF-4E82-B8FE-F95A80A2694D

http://species-id.net/wiki/Tetramorium_triangulatum

Figs 4 [Fig F3] 12

#### Holotype.

Worker, India, Himachal Pradesh, Andretta, 32.036638°N, 76.566532°E, 940m alt., soil core, 19 June 2010, coll. R. Kumar, PUPAC.

#### Paratypes.

15(q), India, Punjab, Patiala, 250m alt., soil core, 13 July 1999, coll. H. Bharti; 25(m), India, Punjab, Patiala, 250m alt., soil core, 13 July 1999, coll. H. Bharti; 1(w), India, Uttarakhand, Assan Barrage, 440m alt., soil core, 10 May 2009, coll. R. Kumar; 46(w), India, Himachal Pradesh, Andretta, 940m alt., soil core, 19 June 2010, coll. R. Kumar; 7(m), India, Himachal Pradesh, Andretta, 940m alt., soil core, 19 June 2010, coll. R. Kumar; 10(q), India, Himachal Pradesh, Andretta, 940m alt., soil core, 19 June 2010; coll. R. Kumar; PUPAC and two paratype will be deposited in BMNH.

#### Worker description.

Measurements. Holotype worker. HL 0.60, HW 0.52, SL 0.38, EL 0.03, WL 0.63, PW 0.37, PSL 0.07, PTL 0.14, PPL 0.20, PTW 0.19, PPW 0.21, PTH 0.21, PPH 0.19, CI 86.67, OI 5.77, SI 73.08, PSLI 11.67, PeNI 51.35, LPeI 66.67, DPeI 135.71, PpNI 56.76, LPpI 105.26, DPpI 105.00, PPI 110.53.

Paratype workers. HL 0.53-0.60, HW 0.45-0.52, SL 0.32-0.38, EL 0.03, WL 0.57-0.63, PW 0.31-0.37, PSL 0.07, PTL 0.12-0.14, PPL 0.17-0.20, PTW 0.16-0.19, PPW 0.19-0.21, PTH 0.19-0.21, PPH 0.16-0.19, CI 84.91-86.67, OI 5.77-6.67, SI 71.11-75.00, PSLI 11.67-13.21, PeNI 50.00-51.61, LPeI 63.16-70.00, DPeI 121.43-135.71, PpNI 56.76-61.76, LPpI 89.47-106.25, DPpI 105.00-123.53, PPI 110.53-123.53 (8 measured).

Head longer than broad, sides weakly convex or almost straight with rounded posterolateral corners, broader posteriorly than anteriorly; posterior head margin straight; clypeus convex with steep apical half; anterior margin of clypeus with a narrow transverse plate like fringe and somewhat impressed medially; mandibles triangular with 7 teeth, masticatory margin of mandibles with large apical and preapical tooth; third tooth slightly smaller than the preapical tooth followed by 4 denticles; frontal lobes weakly developed, frontal area indistinct; antennal scrobes absent; eye small in size, located laterally and below mid-length of head, composed of single ommatidium; antennae slender, 12-segmented; scape not reach to posterior head margin and 0.63× head length; mesosoma slightly longer than head, broader anteriorly than posteriorly, dorsum flat, tapering backwards; pro-mesonotal suture and metanotal groove indistinct; propodeal spine short (PSL 0.07mm), triangular, acute, divergent and slightly longer than propodeal lobes; propodeal lobes triangular and acute; posterior declivity of propodeum short, concave; petiolar node as broad as long in dorsal view; weakly convex dorsum in lateral view; peduncle short, with a large, straight lamella ventrally; postpetiole broader than long, base of first gastral tergite concave behind the postpetiole, anterolateral corners prominent and projecting forward as a pair of blunt teeth or horns which go round the sides of the posterior portion of the postpetiole, gaster oval.

Head longitudinally rugulose, interrugal space somewhat granular, punctured and shiny; frontal carinae very short, ending in front of the level of the eyes; mandibles longitudinally rugulose and finely punctured; clypeus longitudinally carinate and these carinae continued to head sculpture; dorsum of mesosoma longitudinally rugulose and interrugal space somewhat granular, punctured; sides of mesosoma finely rugo-reticulate; petiole and postpetiole mostly smooth with traces of rugosity; propodeal declivity with traces of fine transverse rugosity, base of first gastral tergite longitudinally rugulose; legs smooth except coxae faintly punctured.

Body yellowish brown; whole body coveredwith long and short, erect and suberect pilosity; antennal scapes and hind tibiae with short suberect hairs.

#### Queen description.

Measurements. HL 0.67-0.71, HW 0.62-0.63, SL 0.42-0.44, EL 0.17-0.19, WL 0.94-0.98, PW 0.57-0.59, PSL 0.13-0.14, PTL 0.14-0.19, PPL 0.24-0.25, PTW 0.23-0.27, PPW 0.30-0.32, PTH 0.28-0.30, PPH 0.27-0.30, CI 88.73-92.54, OI 26.98-30.16, SI 67.74-69.84, PSLI 18.31-19.72, PeNI 38.98-45.76, LPeI 50.00-63.33, DPeI 135.29-171.43, PpNI 50.85-54.24, LPpI 83.33-88.89, DPpI 125.00-128.00, PPI 118.52-130.43 (3 measured).

Similar to the worker in structure, sculpture and pilosity except the following characters (besides characters related to wings): eyes large in size, head with three ocelli.

#### Male description.

Measurements. HL 0.50-0.52, HW 0.46-0.48, SL 0.23-0.24, EL 0.23, WL 1.02-1.05, PW 0.66-0.69, PTL 0.14-0.16, PPL 0.21-0.24, PTW 0.21-0.23, PPW 0.27-0.28, PTH 0.23-0.24, PPH 0.23-0.25, CI 92.00-92.31, OI 47.92-50.00, SI 50.00, PeNI 31.82-34.33, LPeI 58.33-69.57, DPeI 131.25-164.29, PpNI 39.13-42.42, LPpI 84.00-104.35, DPpI 112.50-133.33, PPI 117.39-133.33 (3 measured).

Head slightly longer than broad, sides convex, posterior head margin convex, with three ocelli; mandibles with well developed 5 pointed teeth but in few specimens large apical teeth followed by series of denticles; clypeus convex and its anterior margin entire, convex with a narrow transverse plate like fringe and not impressed medially; frontal lobes reduced; antennae 10-segmented; scape short and not reach to posterior head margin and almost 0.46× times head length; apical segment longer and twice of preapical segment, followed by 4 segments as long as broad; then followed by a segment slightly longer than broad; antennal segment attached to pedicel much longer and almost equal to apical segment which then followed by a small segment (smaller than all flagellar segments); antennal scrobes absent; eyes large, convex, situated laterally and more towards lower half of head; pronotum broader than head with rounded anterior lateral angles; mesoscutum and mesoscutellum flat; propodeal dorsal face flat and oblique with vertical declivitous part; propodeal spines absent; propodeal lobes almost rounded; petiole longer, almost 1.4× times its width, with a ventral lamella along its entire length; Postpetiole broader than long; gaster convex, elongate, oval; longer legs.

Head and clypeus longitudinally rugulose and spaces between them punctured; mandibles punctured, frontal carinae continued to the posterior ocelli; dorsum and sides of mesosoma smooth and shiny except propodeum; propodeum finely longitudinally rugulose and punctured; node of petiole, postpetiole, gaster and legs smooth and shiny except few trace of sculpture on sides of node of petiole; wings transparent.

Body yellowish brown with 1^st^ gastral tergite much darker and area of ocelli blackish; body covered with short and long suberect pilosity.

#### Etymology.

The specific epithet refers to the triangular propodeal spines.

#### Ecology.

This species is uncommon in the Shivalik range of the north-western Himalaya and was collected from soil.

#### Remarks.

*Tetramorium triangulatum* sp. n. belongs to the *inglebyi*-species group (Bolton 1977) which is apparently restricted to India and is easily characterized by antennae 12-segmented, appendage of sting triangular or dentiform, frontal carinae absent or very short, not reaching the level of the anterior margin of the eyes, eyes small, reduced to a single ommatidium in *myops*, antennal scrobes absent, base of first gastral tergite strongly concave in dorsal view, the anterolateral angles of the sclerite angular, produced as a short tubercles or tooth on each side of the posterolateral corners of the postpetiole.

This new species is close to *Tetramorium myops* Bolton as both species possess short frontal carinae, minute eyes, peduncle with ventral lamella and an medially impressed anterior clypeal margin. However, this new species can be easily distinguished from *Tetramorium myops* by the following combination of characters: *Tetramorium triangulatum* sp. n. has short propodeal spines (PSL 0.07mm), triangular and divergent, peduncle with a large and straight lamella ventrally, dorsum of mesosoma longitudinally rugulose, base of first gastral tergite longitudinally rugulose, while in case of *Tetramorium myops* the propodeal spines are long and upcurved along their length, the peduncle with a large rounded and convex lamella ventrally, dorsum of mesosoma with longitudinal rugulae and reticulation, base of first gastral tergite with vestiges of superficial sculpture. Some of the other significant characters of *Tetramorium triangulatum* sp. n. which differentiate it from *Tetramorium myops* are: head broader posteriorly with straight sides and SI 71.11-75.00 versus head with convex sides and SI 77.00-83.00 in *Tetramorium myops*.

**Figures 4–6.**
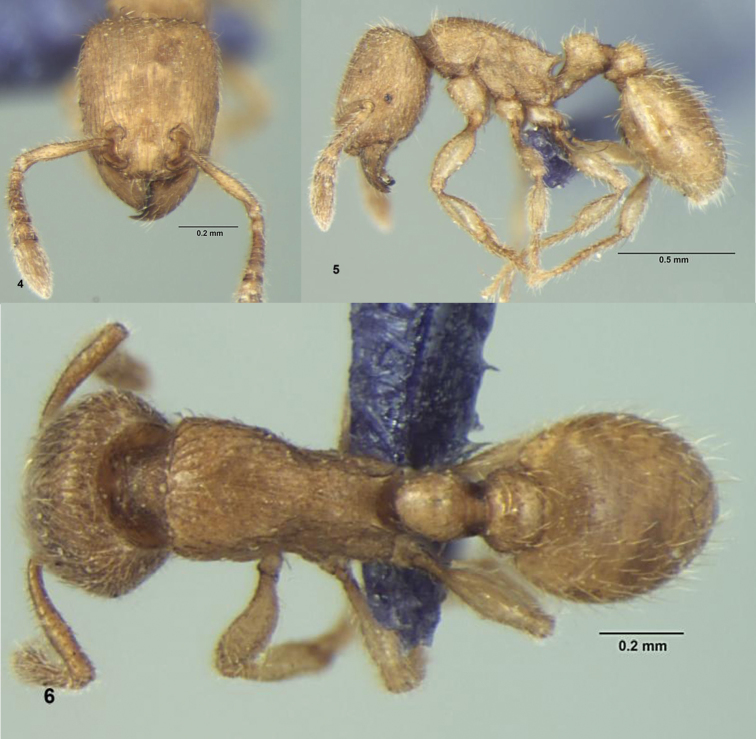
*Tetramorium triangulatum* sp. n., Worker: **4** Head, full-face view **5** Body, lateral view **6** Body, dorsal view.

**Figures 7–9. F3:** *Tetramorium triangulatum* sp. n., Queen: **7** Head, full-face view **8** Body, lateral view **9**  Body, dorsal view.

**Figures 10–12.**
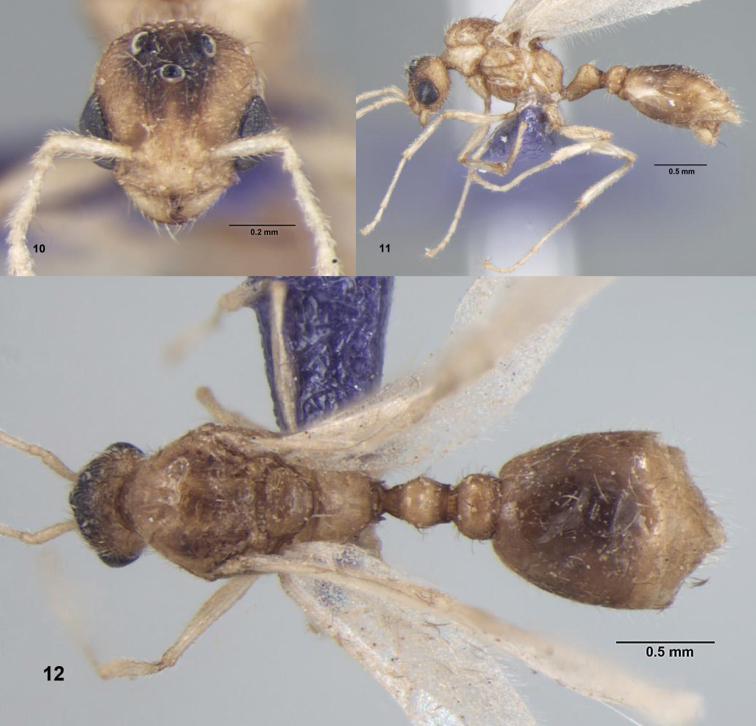
*Tetramorium triangulatum* sp. n., Male: **10** Head, full-face view **11** Body, lateral view **12** Body, dorsal view.

### Tetramorium caldarium

Roger, 1857 new record from India

http://species-id.net/wiki/Tetramorium_caldarium

Figs 13–15

#### Material examined.

11(w), India, Punjab, Patiala, 250m alt., hand picking, 07 April 2011, coll. R. Kumar, PUPAC.

#### Worker description.

Measurements (worker). HL 0.63-0.64, HW 0.53-0.56, SL 0.42-0.45, EL 0.12-0.13, WL 0.66-0.69, PW 0.37-0.38, PSL 0.03-0.05, PTL 0.13-0.14, PPL 0.18-0.20, PTW 0.18-0.19, PPW 0.21-0.23, PTH 0.20-0.21, PPH 0.18-0.19, CI 84.13-87.50, OI 21.82-23.21, SI 76.36-83.02, PSLI 4.76-7.94, PeNI 47.37-51.35, LPeI 61.90-70.00, DPeI 128.57-146.15, PpNI 55.26-62.16, LPpI 94.74-105.56, DPpI 110.53-127.78, PPI 116.67-121.05 (9 measured).

Head longer than broad, sides almost straight, posterolateral corners rounded, posterior head margin shallowly emarginated; clypeus consisting of flat basal half and steep apical half; anterior margin of clypeus entire without median notch; mandibles triangular, with 6 teeth, masticatory margin of mandibles with large apical and preapical tooth; third tooth slightly smaller than the preapical tooth followed by three denticles; frontal lobes weakly developed and elevated laterally, frontal area indistinct; antennal scrobes feeble, indistinct, very little concave and not bordered posteriorly; eye moderate in size, located laterally and almost at mid-length of head, composed of ca. 8 ommatidia in a series along its maximum length; antennae slender, 12-segmented; scape short from posterior head margin by one fourth of its length; mesosoma longer than head, broader anteriorly than posteriorly, dorsum flat and tapers to backward in lateral view; pro-mesonotal suture and metanotal groove indistinct; propodeal teeth small (PSL 0.03-0.05mm), acute, triangular almost equal to its width and propodeal lobes; propodeal lobes broad and roughly triangular in shape; posterior declivity of propodeum short, concave; petiole with a short peduncle, its node as broad as long with anterior and posterior faces parallel, weakly convex dorsum in lateral view; ventrally petiole weakly downcurved along its length; peduncle with a small anteroventral lamella; postpetiole broader than long, gaster oval.

Head feebly longitudinally rugose, interrugal space weakly granular or punctulate; frontal carinae feeble and developed to the level of the midlength of the eye behind which fade out or broken; mandibles weakly longitudinally rugulose; clypeus with a strong mid and two lateral carinae; dorsum of mesosoma weakly granular or punctulate with traces of rugulose sculpture; sides of mesosoma reticulate; petiole and postpetiole faintly rugulose and punctulate; propodeal declivity reticulate, upper half finely transversely rugulose, gaster unsculptured; legs smooth and shiny.

Whole body yellowish brown with gaster darker brown; body with short, erect, stiff, blunt hairs and few scattered pubescence on gaster; antennal scapes and hind tibiae with very short, fine, appressed pubescence.

#### Remarks.

Being tramp, it was collected from a disturbed area with high anthropogenic activities. It is widely distributed in the tropics and subtropics, associated with man and living in hothouses, zoos or other constantly heated buildings (Bolton 1980). It is uncommon in India and has been reported for the first time from India. It belongs to *simillimum-* species group (Bolton 1980, Hita Garcia and Fisher 2011) and is very close to *Tetramorium simillimum* (Smith). From the latter, it can be distinguished by the following combination of characters: frontal carinae developed to the level of the midlength of the eyes behind which they become very weak or broken, or gradually fade out posteriorly, ground sculpture of head is feeble with surfaces dully shiny; antennal scrobes vestigial.

**Figures 13–15.**
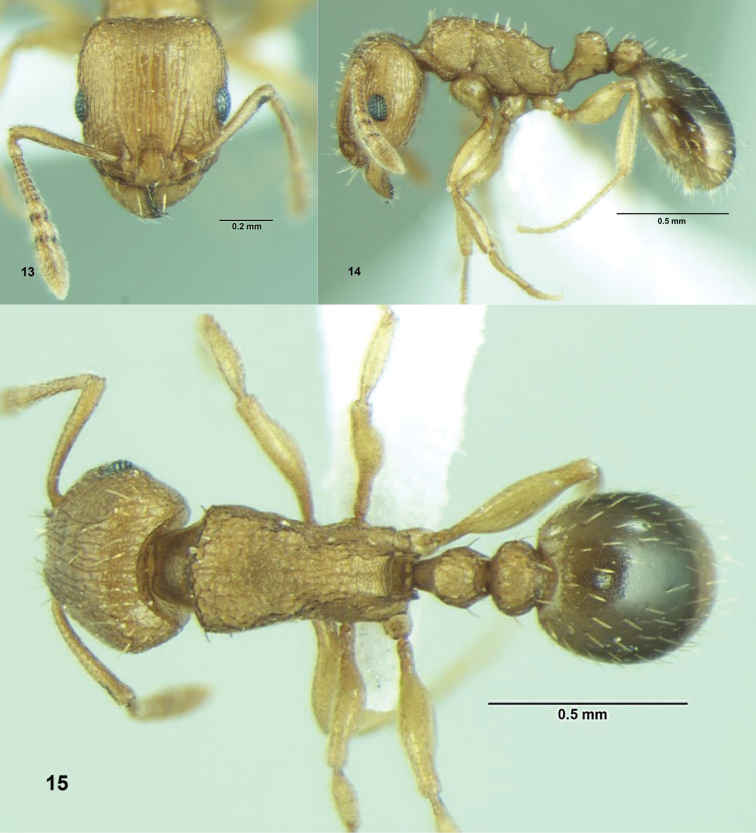
*Tetramorium caldarium* (Roger), Worker: **13** Head, full-face view **14** Body, lateral view **15** Body, dorsal view.

### Tetramorium tonganum

Mayr (1870) new record from India

http://species-id.net/wiki/Tetramorium_tonganum

Figs 16 [Fig F7] 24

#### Material examined.

1(w), India, Himachal Pradesh, Lwasa, 1200m alt., soil core, 07 May 2009; 1(w), India, Uttarakhand, Dehradun, Forest Research Institute, 640m alt., soil core, 17 August 2009; 14(w), India, Himachal Pradesh, Baijnath, 1000m alt., soil core, 17 June 2010; 8(m), India, Himachal Pradesh, Baijnath, 1000m alt., soil core, 17 June 2010; 13(q), India, Himachal Pradesh, Baijnath, 1000m alt., soil core, 17 June 2010; 3(q), India, Himachal Pradesh, Andretta, 940m alt., soil core, 20 June 2010; 8(m), India, Himachal Pradesh, Andretta, 940m alt., soil core, 20 June 2010; 28(w), India, Himachal Pradesh, Andretta, 940m alt., soil core, 20 June 2010; 14(w), India, Mandi, Himachal Pradesh, 800m alt., soil core, 27 June 2010; coll. R.Kumar; PUPAC.

#### Worker description.

Measurements (worker).HL 0.62-0.66, HW 0.56-0.60, SL 0.46-0.48, EL 0.14, WL 0.69-0.75, PW 0.42-0.46, PSL 0.07-0.10, PTL 0.16-0.17, PPL 0.20-0.23, PTW 0.21-0.26, PPW 0.23-0.26, PTH 0.20-0.21, PPH 0.20-0.23, CI 90.32-92.19, OI 23.33-25.00, SI 76.67-85.71, PSLI 11.29-15.63, PeNI 50.00-56.52, LPeI 80.00-85.00, DPeI 131.25-152.94, PpNI 52.27-56.52, LPpI 86.96-115.00, DPpI 100.00-130.00, PPI 100.00-109.52 (7 measured).

Head slightly longer than broad, sides almost straight with rounded posterolateral corners, slightly broader posteriorly than anteriorly; posterior head margin straight with shallow median notch; clypeus convex with steep apical half; anterior margin of clypeus entire without median notch; anterior margin of clypeus with a narrow transverse plate like fringe and having convex anterior margin; mandibles triangular, masticatory margin of mandibles with 7 teeth, large apical and preapical teeth; third tooth slightly smaller than the preapical tooth, fourth tooth smaller than the following teeths; frontal lobes weakly developed and slightly elevated laterally, frontal area distinct; antennal scrobes shallow; eye moderate in size, located laterally and almost at mid-length of head, composed of ca. 38-40 ommatidia; antennae slender, 12-segmented; scape long and just fail to reach posterior head margin; mesosoma longer than head, broader anteriorly than posteriorly, dorsum convex; pro-mesonotal suture and metanotal groove indistinct; propodeal spine longer (PSL 0.07-0.10mm) than propodeal lobes, acute, divergent, directed upwards; propodeal lobes broadly triangular; posterior declivity of propodeum short, concave; petiole with a long peduncle, node subglobular in dorsal view; ventrally petiole downcurved along its length, peduncle with an antero-ventral minute blunt teeth; petiole and postpetiole almost equally broader; gaster oval.

Head longitudinally rugose with few cross-meshes upto vertex, posteriorly reticulate, interrugal space punctured and shiny; frontal carinae more conspicuous than other cephalic sculpture and reaching to posterior head margin; mandibles longitudinally rugulose; clypeus longitudinally rugulose; dorsum and sides of mesosoma reticulate-rugulose; petiolar node with weak rugosity; postpetiole unsculptured; propodeal declivity and gaster smooth and shiny, legs smooth except coxae with punctures.

Body yellowish brown while gaster somewhat darker; body coveredwith suberect abundant hairs of varying length; antennal scapes and hind tibiae with decumbent short pubescence.

#### Queen description.

Measurements (queen). HL 0.69-0.70, HW 0.64-0.66, SL 0.50-0.52, EL 0.20-0.21, WL 1.00-1.02, PW 0.64-0.66, PSL 0.12-0.13, PTL 0.16-0.17, PPL 0.27-0.28, PTW 0.31-0.32, PPW 0.31-0.32, PTH 0.27, PPH 0.27-0.28, CI 92.75-94.29, OI 30.77-31.82, SI 78.13-80.00, PSLI 17.14-18.84, PeNI 48.44-48.48, LPeI 59.26-62.96, DPeI 182.35-193.75, PpNI 48.44-50.00, LPpI 96.43-103.70, DPpI 114.29-114.81, PPI 100-103.23 (3 measured).

Similar to the worker in structure, sculpture and pilosity except the following characters (besides characters related to wings ): eyes large in size and with ca.10-12 ommatidia in a series along its maximum length, head with three ocelli, dorsum of mesosoma flat, propodeal spines slightly longer, petiolar node transverse, broad, pronotum reticulate; mesoscutum, mesoscutellum, anepisternum and sides of propodeum longitudinally rugulose; katepisternum smooth, base of propodeum transversally rugulose; petiolar node rugo-reticulate; postpetiole smooth with traces of sculpture on sides, coxae with faint transverse rugulae.

#### Male description.

Measurements. HL 0.55-0.56, HW 0.52-0.53, SL 0.27-0.30, EL 0.28-0.31, WL 1.07-1.12, PW 0.69-0.74, PTL 0.16-0.19, PPL 0.25-0.27, PTW 0.24-0.27, PPW 0.25-0.28, PTH 0.20-0.21, PPH 0.23-0.27, CI 92.86-94.64, OI 53.85-59.62, SI 51.92-56.60, PeNI 34.78-36.99, LPeI 80.00-95.00, DPeI 142.11-158.82, PpNI 36.23-38.36, LPpI 100.00-117.39, DPpI 92.59-112.00, PPI 103.70-104.17 (3 measured).

Head slightly longer than broad, sides convex, posterior head margin straight or weakly convex, with three ocelli; mandibles with well developed 6 pointed teeth; apical tooth large followed by 5 short teeth; clypeus convex and its anterior margin entire, convex with a narrow transverse plate like fringe and not impressed medially; frontal lobes reduced; antennae 9-segmented; scape short and not reach to posterior head margin and almost 0.5x times head length; apical segment longer and more than twice of preapical segment; second flagellar segment longer than scape (0.34mm); antennal scrobes absent; eyes large, convex, situated laterally and more towards lower half of head; pronotum broader than head with rounded anterior lateral angles; mesoscutum convex and mesoscutellum flat; propodeal dorsal face flat and oblique with vertical declivitous part; propodeal spines absent; propodeal lobes almost rounded; petiole longer, almost 1.4× times its width, petiolar node subglobular and medially sulcate shallowly; Postpetiole slightly broader than long; gaster convex, broadly oval; longer legs.

Head and clypeus longitudinally rugulose and spaces between them punctured; mandibles smooth with traces of sculpture, frontal carinae continued to the each side of anterior ocellus; dorsum and sides of mesosoma mostly smooth with traces of sculpture and shiny except propodeum; propodeum finely longitudinally rugulose and punctured; node of petiole longitudinally rugulose except median sulcate region; postpetiole, gaster and legs smooth and shiny; wings transparent.

Body yellowish brown except 1^st^ gastral tergite brownish and area between ocelli blackish; body covered with short and long suberect pilosity.

#### Remarks.

This species has been found to be widespread in the Shivalik range of the north-western Himalaya and represents a new record for India. Its male caste has been described for the first time. This species belongs to the *tonganum*-species group (Bolton 1977) and resembles *Tetramorium difficile* Bolton. It can be distinguished from the latter due to larger size, relatively longer antennal scape, longer legs, broadly rounded pronotal corners and propodeum with long spine.

**Figures 16–18.**
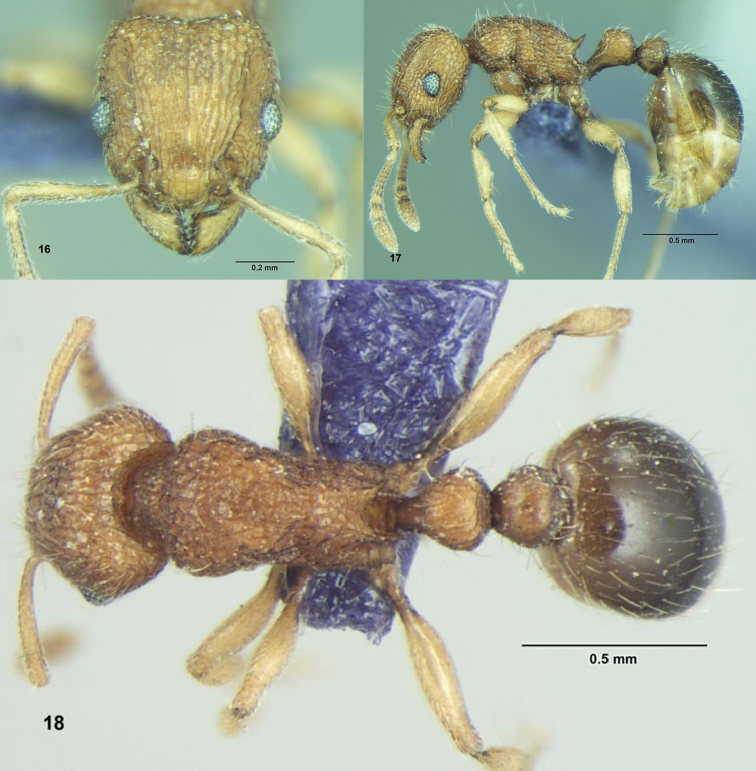
*Tetramorium tonganum* Mayr, Worker: **16** Head, full-face view **17** Body, lateral view **18** Body, dorsal view.

**Figures 19–21. F7:**
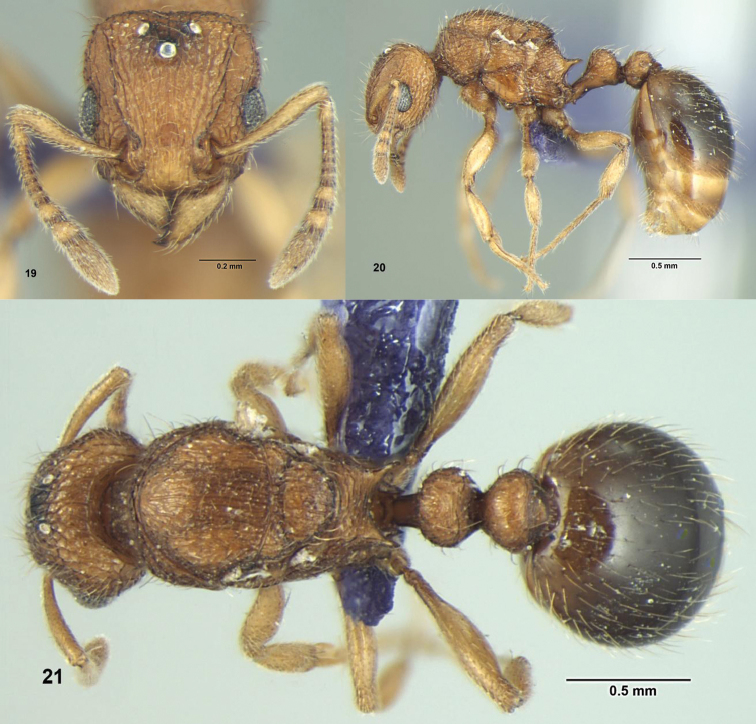
*Tetramorium tonganum* Mayr, Queen: **19** Head, full-face view; **20** Body, lateral view **21** Body, dorsal view.

**Figures 22–24.**
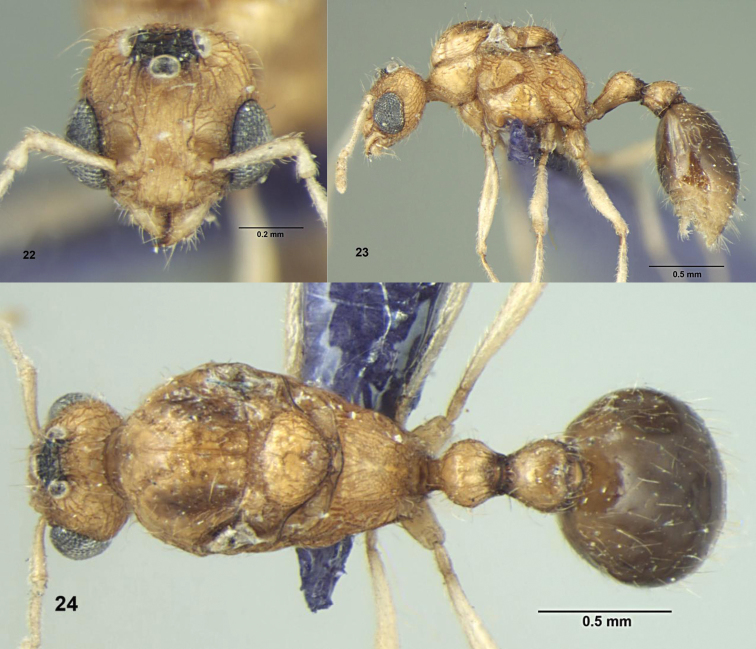
*Tetramorium tonganum* Mayr, Male: **22** Head, full-face view; **23** Body, lateral view **24** Body, dorsal view.

### Tetramorium urbanii

Bolton, 1977

http://species-id.net/wiki/Tetramorium_urbanii

Figs 25–27

#### Material examined.

2(w), India, Shillong, 20 May 2003, hand picking, coll. H. Bharti.

#### Worker description.

Measurements (worker). HL 0.85-0.87, HW 0.74- 0.75, SL 0.70, EL 0.16, WL 1.05, PW 0.60, PSL 0.06, PTL 0.28-0.30, PPL 0.32-0.34, PTW 0.28, PPW 0.35-0.36, PTH 0.32-0.34, PPH 0.35, CI 85.06-88.24, OI 21.33-21.62, SI 93.33-94.59, PSLI 6.90-7.06, PeNI 46.67, LPeI 82.35-93.75, DPeI 93.33-100.00, PpNI 58.33-60.00, LPpI 91.43-97.14, DPpI 105.88-109.38, PPI 125.00-128.57 (2 measured).

Head longer than broad, sides almost straight, rounded posterolateral corners, posterior head margin straight, very feebly indented medially; clypeus consisting of slightly convex basal half and steep apical half, without anteromedian indentation; anterior margin of clypeus with a narrow transverse plate like fringe and having convex anterior margin; mandibles triangular, with 7 teeth, masticatory margin of mandibles with large apical and preapical tooth; third tooth slightly smaller than the preapical tooth followed by four denticles; frontal lobes weakly developed and elevated laterally; frontal area deep, forming concavity behind clypeus, broader than long; antennal scrobe distinct, strongly margined dorsally by the frontal carina; eye moderate in size, located laterally and at mid-length of head, composed of ca. 9-10 ommatidia in a series along its maximum length; antennae slender, 11-segmented; scape reaching to posterolateral corners of head; mesosoma longer than head, broader anteriorly than posteriorly, dorsum convex in lateral view; pro-mesonotal suture and metanotal groove indistinct; propodeal teeth small (PSL 0.06mm), triangular, almost equal to its width and propodeal lobes; propodeal lobes narrowly rounded; posterior declivity of propodeum short, slightly concave, separated from dorsum by a strong transverse carina; petiole with a short peduncle, its node longer than broad with convex dorsum, broader behind than front; ventrally petiole downcurved along its length; postpetiole slightly broader than long, gaster oval.

Head longitudinally rugose with few cross meshes; interrugal space punctured and somewhat shiny; frontal carinae strongly developed, almost straight, divergent at eye level, running back almost to the posterior head margin; antennal scrobes feebly sculptured; mandibles longitudinally striate; clypeus longitudinally rugulose; promesonotal dorsum mostly unsculptured smooth and shiny with traces of rugulose sculpture towards the sides and posteriorly; propodeum reticulate, sides of mesosoma longitudinally rugulose; petiole and postpetiole longitudinally rugulose; propodeal declivity and gaster smooth and shiny; legs smooth.

Whole body blackish brown except mandibles, antennae and legs yellowish brown. Whole body coveredwith abundant, long, erect and short subdecumbent pilosity; antennal scapes and hind tibiae with short subdecumbent hairs.

#### Remarks.

*Tetramorium urbanii* belongs to the *tortuosum*- group (Bolton 1977). It has very short propodeal spines and moderately long scapes, these characters clearly separate it from the rest of the *tortuosum*- group species. This species was earlier reported from Bhutan and represents a first record from India.

**Figures 25–27.**
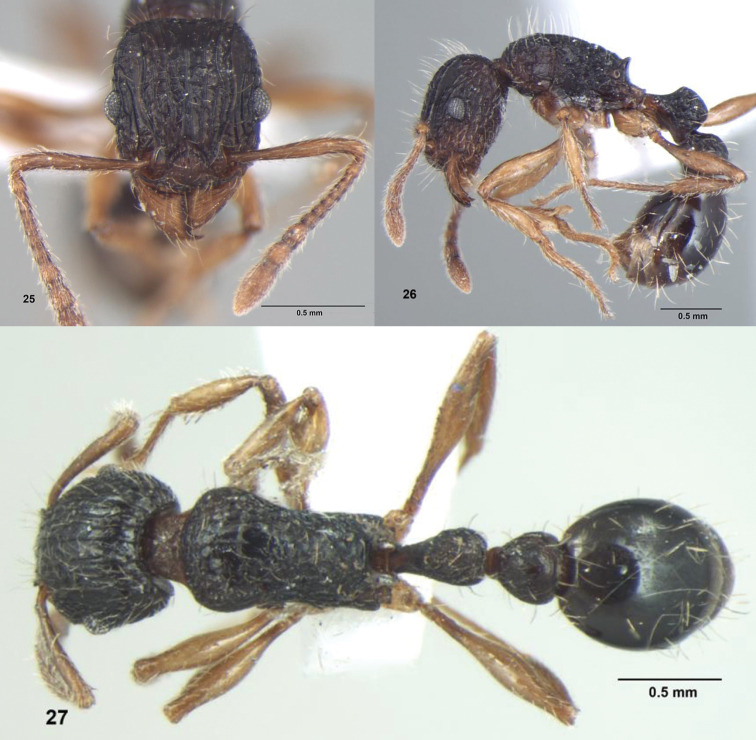
*Tetramorium urbanii* Bolton, Worker: **25** Head, full-face view; **26** Body, lateral view **27** Body, dorsal view.

##### Key to the Indian species of *Tetramorium* based on the worker caste (modified from Bolton 1976 and 1977)

**Table:** 

1	The species with numerous branched hairs (bifid, trifid or quadrifid and in a few species a mixture of two or more of these may be present)	2
–	The species with only simple hairs	9
2	Antennae with 10 segments, propodeum unarmed	*Tetramorium decamerum* (Forel)
–	Antennae with 12 segments, propodeum with a pair of spines or teeth	3
3	Gaster cordate (heart) in shape	*Tetramorium cordatum* Sheela and Narendran
–	Gaster non cordate	4
4	Basal one-quarter to two-thirds of first gastral tergite distinctly sculptured with striation, puncturation or a combination of both; node of petiole in dorsal view strongly antero-posteriorly compressed, transverse, distinctly broader than long	*Tetramorium walshi* (Forel) (part)
–	Basal portion of first gastral tergite not sculptured, the entire sclerite smooth and shiny; node of petiole variable in shape	5
5	Node of petiole in dorsal view strongly antero-posteriorly compressed, transverse, distinctly broader than long	*Tetramorium walshi* (Forel) (part)
–	Node of petiole in dorsal view not antero-posteriorly compressed, not transverse, generally as long as broad or very slightly broader than long	6
6	Dorsal surface of hind tibiae viewed from in front or behind with abundant short, curved hairs of approximately uniform length which are much shorter than the maximum tibial width; these hairs characteristically forming a close mat above the tibial surface. Antennal scrobes vestigial, without an acute dorsal margin	*Tetramorium rossi* (Bolton)
–	Dorsal surface of hind tibiae viewed from in front or behind with numerous long hairs of varying length, the longest of them at least subequal to the maximum tibial width; these hairs never forming a close mat above the tibial surface. Antennal scrobes developed with a marked dorsal margin	7
7	First gastral tergite with all hairs simple	*Tetramorium coonoorense*Forel
–	First gastral tergite with at least a few bifid or trifid hairs present	8
8	Mandibles smooth with scattered punctures, not longitudinally striate; HW 0.76–0.82mm; first gastral tergite with trifid hairs on basal half	*Tetramorium obesum* Andre
–	Mandibles longitudinally striate, the striation sometimes indistinct or absent in small specimens; HW 0.52–0.70mm; first gastral tergite basally with a mixture of simple and bifid hairs, trifid hairs usually completely absent	*Tetramorium lanuginosum* Mayr
9	Antennae with 11 segments	10
–	Antennae with 12 segments	14
10	Mandibles smooth with scattered pits, not striate. Small species, SL 0.42-0.46mm.	*Tetramorium smithi* Mayr
–	Mandibles longitudinally striate; usually this sculpture coarse and distinct but if faint then species much larger, SL 0.58mm	11
11	Propodeum armed with a pair of short triangular teeth which are only slightly longer than their basal width and only marginally longer than the propodeal lobes	*Tetramorium urbanii* Bolton
–	Propodeum armed with a pair of long spines which are much longer than their basal width and considerably longer than the propodeal lobes	12
12	Postpetiole punctulate propodeal lobes rounded, not acute at apex	*Tetramorium keralense* Sheela and Narendran
–	Dorsum of postpetiole unsculptured, smooth and shiny, propodeal lobes triangular and acute	13
13	Dorsal mesosoma with rugulae tending to be effaced and replaced by shiny areas, HL 0.82–0.92mm, HW 0.78–0.86mm, SL 0.70–0.80mm, postpetiole unsculptured; entire body uniform dark brown or blackish brown	*Tetramorium tortuosum* Roger
–	Dorsal mesosoma reticulate rugose, HL 0.74–0.80mm, HW 0.66–0.70mm, SL 0.58–0.64mm, sides of postpetiole with rugulose sculpture; colour light brown with gaster darker than head and mesosoma	*Tetramorium belgaense* Forel
14	Species larger in size, body total length 5-6mm	*Tetramorium beesoni* (Mukerjee)
–	Species total length less than 5mm	15
15	Posterior head margin emarginated	*Tetramorium meghalayense* Bharti
–	Posterior head margin either emarginated or non emarginated	16
16	Lamelliform appendage of sting dentiform, triangular or pennant-shaped and projecting at an angle from the shaft, antennal scrobes either present or absent; frontal carinae either short or long	17
–	Lamelliform appendage of sting linear and spatulate, continuing the line of the shaft, antennal scrobes absent, frontal carinae short, ending before level of eyes	*Tetramorium fergusoni* Forel
17	Frontal carinae short terminating at or in front of the level of the eyes; dorsum of head variably sculptured	18
–	Frontal carinae long, projecting back beyond the level of the eyes. If the carinae fade out just behind the level of the eyes then the dorsum of the head is regularly, very densely longitudinally rugose or evenly sulcate	22
18	Eyes minute with only a single ommatidium. Peduncle of petiole with large anteroventral lamella	19
–	Eyes large with five or more ommatidia. Peduncle of petiole without a large anteroventral lamella	20
19	Anterior clypeus with distinct median impression, dorsal mesosoma reticulate-rugose and propodeal spines relatively long upcurved along their length	*Tetramorium myops* Bolton
–	Anterior clypeus without any median impression, dorsal mesosoma longitudinally rugulose, and propodeal spines relatively short (PSL 0.07mm), triangular	*Tetramorium triangulatum* sp. n.
20	Head without any reticulate or rugoreticulate structure, petiole and postpetiole finely sculptured with a smooth median area or smooth median longitudinal strip on dorsum, propodeal spines not upcurved	*Tetramorium caespitum*( Linnaeus)
–	Head with reticulate sculpture on its posterior region or sides, petiole and postpetiole mostly unsculptured, propodeal spines upcurved	21
21	Petiole node in dorsal view about as long as broad. Median portion of clypeus abruptly downcurved so that its anterior one-third is vertical and separated by a marked angle from the more posterior portion	*Tetramorium inglebyi* Forel
–	Petiole node in dorsal view much broader than long. Median portion of clypeus evenly convex in its anterior half	*Tetramorium elisabethae* Forel
22	Basal half or more of first gastral tergite sculptured, usually strongly so, with rugosity, dense striation, dense puncturation or a combination of these	23
–	Basal half of first gastral tergite unsculptured or at most with sparse, short, regular basigastral costulae or a few pits from which hairs arise	24
23	Anterolateral angles of first gastral tergite projecting forward as a pair of blunt teeth or tubercles; basal half of first gastral tergite and sternite strongly rugulose	*Tetramorium rugigaster* Bolton
–	Anterolateral angles of first gastral tergite angular but not produced into teeth or tubercles, first gastral tergite and sternite entirely finely reticulate	*Tetramorium malabarense* Sheela and Narendran
24	With the gaster in dorsal view the lateral corners of the base extended forward as a pair of horns which surround the posterior portion of the postpetiole	25
–	With the gaster in dorsal view the lateral corners of the base rounded or sometimes bluntly angular, but never extended forward as a pair of horns which surround the posterior portion of the postpetiole	26
25	Anterolateral angles of first gastral tergite projecting forward as a pair of blunt teeth or horns which go round the sides of the posterior portion of the postpetiole; propodeal lobes elongate triangular and acute	*Tetramorium mixtum* Forel
–	Anterolateral angles of first gastral tergite projecting forward as a pair of acute teeth which go round the sides of the posterior portion of the postpetiole; propodeal lobes subtriangular with rounded tip	*Tetramorium sentosum* Sheela and Narendran
26	Anterior clypeal margin with the median portion convex and notched or sharply indented medially	27
–	Anterior clypeal margin with the median portion entire, varying from convex to broadly and shallowly concave, but never notched or sharply indented medially	31
27	Mandibles sculptured with fine, dense striation or shagreening, occasionally the striation faint	28
–	Mandibles completely smooth and shiny except for scattered hair-pits	30
28	Colour uniform dark brown to blackish brown. Petiole in profile with a narrow anterior peduncle, a short anterior face which curves into the long convex dorsum and a posterior face which is much higher than the anterior. In dorsal view the node is usually slightly longer than broad	*Tetramorium pacificum* Mayr (part)
–	Colour yellow brown to orange-brown, sometimes with the gaster darker brown. Rarely entirely coloured dark brown approaching that of *pacificum*, but in this case the petiole of different shape	29
29	Longest hairs projecting dorsally from frontal carinae behind the level of the antennal insertions shorter than the maximum diameter of the eye. Gaster always much darker in colour than alitrunk and head, contrasting strongly with them. Petiole node in profile roughly square, the dorsum not sloping upwards posteriorly, the anterodorsal and posterodorsal angles approximately on a level. Propodeal spines moderately long, varying from more or less straight to slightly upcurved along their length	*Tetramorium bicarinatum* (Nylander)
–	Longest hairs projecting dorsally from frontal carinae behind the level of the antennal insertions longer than the maximum diameter of the eye. Gaster usually same colour as alitrunk and head, only rarely noticeably darker. Petiole node in profile with the dorsum sloping upwards posteriorly, so that the posterodorsal angles is on a slightly higher level than the anterodorsal. Propodeal spines usually short, elevated but more or less straight, not upcurved along their entire length	*Tetramorium indicum* Forel
30	Petiole in profile with a short anterior face which curves into the long convex dorsum and a posterior face which is slightly higher than the anterior, OI 25.81(eyes comparatively larger in size 0.24mm), CI 100, SI 67	*Tetramorium petiolatum* Sheela and Narendran
–	Petiole in profile with a short anterior face which curves into the long convex dorsum and a posterior face which is much higher than the anterior, OI 20.59–25 (eyes comparatively smaller 0.18-0.21mm), CI 83-90, SI 79-87	*Tetramorium pacificum* Mayr (part)
31	Spaces between rugulose sculpture on entire dorsum of head (and often dorsal alitrunk) completely filled by a dense and very conspicuous reticulate-puncturation so that the surface appears dull, mat and very granular, the punctulate sculpture often as conspicuous as the rugulae	32
–	Spaces between rugulose sculpture on dorsum of head either smooth or with superficial faint or vestigial sculpture so that the surface appears mostly or entirely shiny and largely or partially smooth, the punctulate sculpture never as conspicuous as the rugulae	33
32	Frontal carinae strongly developed throughout their length, sinuate, running unbroken almost to the posterior head margin and surmounted throughout their length ba a narrow raised rim or flange. The whole of the frontal carinae much more strongly developed than the remaining cephalic rugulae. Ground sculpture of head between frontal carinae strongly granular or reticulate- punctulate, the surface matt. Antennal scrobes shallow but broad and conspicuous	*Tetramorium simillimum* (Smith)
–	Frontal carinae feebly developed, weakly or not sinuate, most strongly developed to level of midlength of eye behind which they become very weak or broken, or gradually fade out posteriorly; not surmounted by a raised rim or flange beyond the level of the midlength of the eye, behind which the carinae are no stronger than the remaining cephalic rugulae. Ground sculpture of head more feeble than above, the surfaces dully shiny. Antennal scrobes vestigial	*Tetramorium caldarium* (Roger)
33	Dorsal surface of hind tibiae with decumbent or appressed pubescence only or with very short hairs which are curved through 90° at the base so that the apical portion of the hairs are nearly flush with the surface; erect or suberect hairs or erect pubescence completely absent from the outer tibial surface, SI ≥ 74	34
–	Dorsal surface of hind tibiae with conspicuous suberect hairs, SI ≤ 67.00	*Tetramorium shivalikense* sp. n.
34	Antennal scapes relatively longer, SI 87-91, colour uniform black	*Tetramorium christiei* Forel
–	Antennal scapes relatively shorter, SI 74-86, colour yellowish brown or dark brown	35
35	Peduncle of petiole in profile short and straight, not downcurved along its length from node to insertion nor passing through a rounded angle at about its midlength. Propodeal lobes bluntly rounded	*Tetramorium salvatum* Forel
–	Peduncle of petiole downcurved along its length. Propodeal lobes triangular	36
36	Propodeal spines short and about the size of triangular propodeal lobes, SI 74-79; EL 0.09mm and body dark brown with a reddish tinge	*Tetramorium barryi* Mathew
–	Propodeal spines longer than the broadly triangular propodeal lobes, SI 80-87, EL 0.14–0.16mm, colour varying from yellowish brown to mid brown	*Tetramorium tonganum* Mayr

## Supplementary Material

XML Treatment for Tetramorium shivalikense

XML Treatment for Tetramorium triangulatum

XML Treatment for Tetramorium caldarium

XML Treatment for Tetramorium tonganum

XML Treatment for Tetramorium urbanii
